# Evidence for a Complex Mosaic Genome Pattern in a Full-length Hepatitis C Virus Sequence

**DOI:** 10.4137/ebo.s1038

**Published:** 2008-10-30

**Authors:** R.S. Ross, J. Verbeeck, S. Viazov, P. Lemey, M. Van Ranst, M. Roggendorf

**Affiliations:** 1 Institute of Virology, National Reference Centre for HCV, Essen University Hospital, University of Duisburg-Essen, Essen, Germany; 2 Laboratory of Clinical Virology, Department of Microbiology and Immunology, Rega Institute for Medical Research, University of Leuven, Leuven, Belgium

**Keywords:** flaviviruses, hepatitis C virus, sequence AY651061, recombination, recombination analysis software, phylogeny programs

## Abstract

The genome of the hepatitis C virus (HCV) exhibits a high genetic variability. This remarkable heterogeneity is mainly attributed to the gradual accumulation of mutational changes, whereas the contribution of recombination events to the evolution of HCV remains controversial so far. While performing phylogenetic analyses including a large number of sequences deposited in the GenBank, we encountered a full-length HCV sequence (AY651061) that showed evidence for inter-subtype recombination and was, therefore, subjected to a detailed analysis of its molecular structure. The obtained results indicated that AY651061 does not represent a “simple” HCV 1c isolate, but a complex 1a/1c mosaic genome, showing five putative breakpoints in the core to NS3 regions. To our knowledge, this is the first report on a mosaic HCV full-length sequence with multiple breakpoints. The molecular structure of AY651061 is reminiscent of complex homologous recombinant variants occurring among other members of the flaviviridae family, e.g. GB virus C, dengue virus, and Japanese encephalitis virus. Our finding of a mosaic HCV sequence may have important implications for many fields of current HCV research which merit careful consideration.

## Introduction

The hepatitis C virus (HCV) is a single-stranded RNA pathogen belonging to the genus Flavivirus in the flaviviridae family (Garnier et al. 2002). Six major HCV genotypes and almost 80 confirmed or at least provisionally assigned subtypes have been identified (Simmonds et al. 2005), generally showing a distinct geographic distribution (Lavanchy and MacMahon, 2000; Weck, 2005). HCV exhibits a high genetic variability. This remarkable heterogeneity is mainly attributed to the gradual accumulation of mutational changes, primarily due to the error-prone nature of the RNA-dependent RNA-polymerase (Simmonds, 2004; Simmonds et al. 2005). The contribution of recombination events to the evolution of HCV, however, remains controversial. Researchers paid considerable attention to the identification and characterisation of possible HCV recombinants after a first mosaic HCV genome had been described in 2002 (Kalinina et al. 2002). Consequently, during the last six years the occurrence of additional inter-genotypic (Kageyama et al. 2006; Noppornpanth et al. 2006; Legrand-Abravanel et al. 2007), inter-subtype (Colina et al. 2004), or inter-quasispecies (Moreno et al. 2006) recombinant variants with yet unknown replicative and clinical potentials were reported worldwide. In chimpanzees, inoculated simultaneously with HCV subtypes 1a, 1b, 2a, and 3a, recombination between the different genomes was also noted (Gao et al. 2007). Although multiple recombination events were reported for other members of the flaviviridae family (Twiddy and Holmes, 2003), we are not aware of any such observations in HCV. In this communication, we therefore present for the first time evidence for numerous inter-subtype breakpoints in an HCV full-length sequence.

## Materials and Methods

During a survey of approximately 1,200 partial HCV core sequences retrieved from GenBank (http://www.ncbi.nlm.nih.gov/Genbank/index.html. Last accessed August 6, 2008), we encountered several ambiguous HCV genotype and subtype assignments (Ross et al. 2008). Among these deviating HCV isolates was a full-length sequence that had been deposited in GenBank under the accession number AY651061. The details of the amplification and cloning procedures used to generate this particular sequence can be inferred from the recent patent US7348011B2 (available at: http://depatisnet.dpma.de/. Last accessed August 6, 2008). In brief, 11 overlapping nucleotide fragments covering the entire genome were amplified by RT-PCR from a serum sample of an Indian patient with chronic HCV infection. After purification of the amplicons from the gel, the fragments were inserted into a pET21 (+) vector. Subsequently, competent E. coli BL21 (DE3) cells were transformed with the obtained plasmid DNA and selected on a LB agar plate with antibiotic and ITPG/X-gal. Various clones expressing high levels of the inserted HCV fragments were chosen for inoculation of LB medium. Plasmid DNA was prepared by an alkaline lysis method. All expanded clones were digested to excise the respective HCV fragments, which were subjected to confirmatory sequencing. The multiple sequences thus obtained for the different regions of the HCV genome were joined by Chromas and Chromas-pro software. Before submission to the GenBank, the entire genome was also cloned and sequenced. AY651061 was subsequently classified as a subtype 1c variant.

Since our analyses based on nucleotides 461–676 (numbering according to Choo et al. 1991) of AY651061 consistently indicated a clustering with genome fragments from HCV subtype 1a and not 1c variants, this phylogenetic incongruence prompted us to carry out a more detailed investigation of this specific strain. First, we performed maximum likelihood (ML) analyses of the core, E1, E2, p7, NS2 and NS3 regions of AY651061 and of HCV reference strains from GenBank by using Paup* v. 4.0 (Swafford, 2008). For each genomic region, the evolutionary model was selected by Modeltest 3.7 (available at: http://darwin.uvigo.es/software/modeltest.html. Last accessed August 6, 2008) (Posada and Crandall, 1998). Phylogenies were estimated by an extensive ML approach with nucleotide substitution models and rate heterogeneity parameters (proportion of invariable sites and alpha shape of the discredited gamma distribution) as determined by the program. Bootstrap analysis (5,000 replicates) was performed using the neighbour-joining (NJ) method. Next, putative recombination events and corresponding breakpoints were identified with SimPlot, v. 3.5.1 (available at: http://sray.med.som.jhmi.edu/SCRoftware/simplot/. Last accessed August 6, 2008) (Lole et al. 1999). The window width and the step size were set at 400 bp and 20 bp respectively. Bootscanning was performed with AY651061 as a query sequence. Finally, ML trees of the genome fragments between the identified breakpoints were reconstructed by using Paup*, v. 4.0. Also for these tree reconstructions, the exhaustive ML analysis was conducted with the model settings as selected by Modeltest 3.7, and NJ bootstrap analysis (5,000 replicates) was performed.

## Results

**Figure 1:**
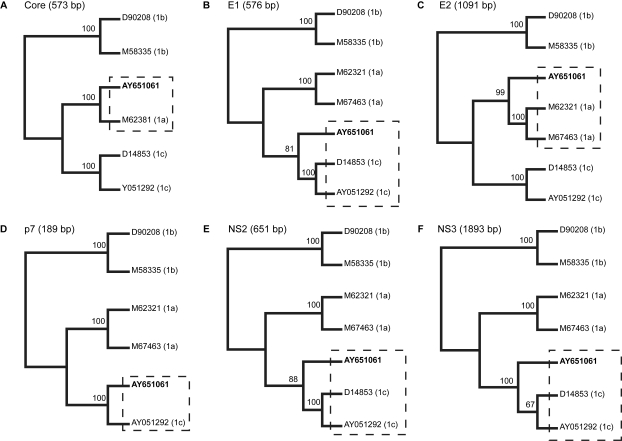
Phylogenetic trees based on maximum likelihood analysis for the core (A) to NS3 (F) regions of AY651061 (represented in bold) and representative HCV subtype 1a, 1b and 1c sequences retrieved from the GenBank database. Identical sequences were removed from the alignment file for the ML analysis of each separate region. The numbers at the nodes of the trees represent bootstrap values (only values of 65 or above are shown). The clusters harbouring the AY651061 strain are indicated by dashed rectangles.

**Figure 2:**
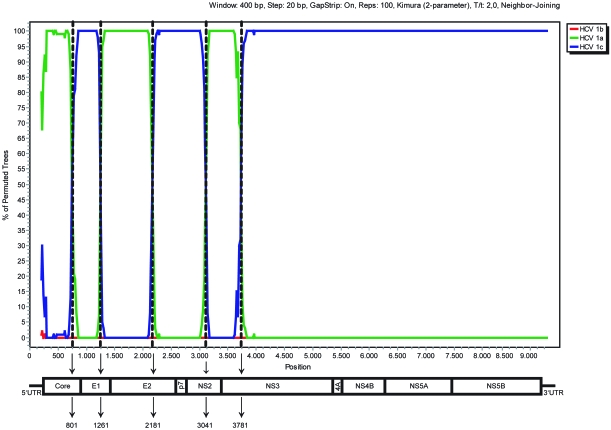
Plot created by the BootScan option of SimPlot, v. 3.5.1. AY651061 was chosen as query sequence and was compared with consensus sequences representing subtypes 1a, 1b and 1c that were obtained by grouping several reference sequences from GenBank. The y-axis represents the number of permutated trees using a sliding window of 400 bp, with a step size of 20 bp. Vertical dashed lines indicate the inter-subtype recombination breakpoints identified by bootscanning analysis. At the bottom, a schematic representation of the HCV genome is shown.

**Figure 3:**
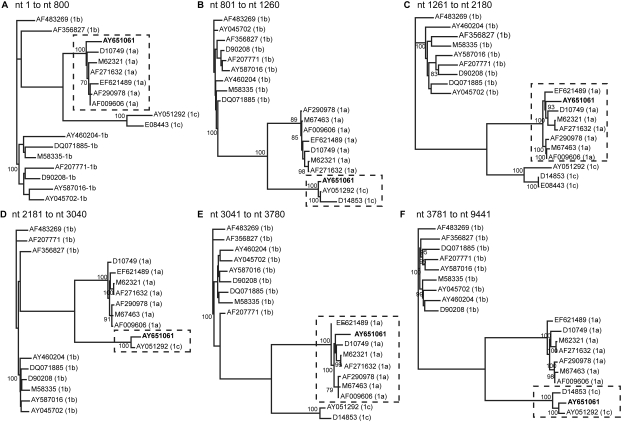
ML phylogenetic trees (A–F) based on the nucleotide fragments between the breakpoints that were identified by SimPlot analysis. AY651061 is represented in bold. Identical sequences were removed from the alignment file for the ML analysis of each separate region. Bootstrap values of 70 or above are shown. The clusters harbouring the AY651061 strain are indicated by dashed rectangles.

Our initial observation on a 216 bp core fragment of AY651061 was corroborated by further phylogenetic analyses of the E1 to NS3 regions of this particular viral strain. As shown in Figure 1, the subgenomic AY651061 sequences form a phylogenetic cluster with HCV 1a variants in the core and E2 regions (Figs. 1A and 1C) but were more similar to HCV 1c isolates in the E1, p7, NS2 and NS3 regions (Figs. 1B, 1D–F), suggesting a mosaic structure. The phylogenetic clustering is supported by high bootstrap values for each of the examined regions. Bootscanning analysis using the approach of a “sliding window” implemented in SimPlot indicated five putative breakpoints (nts 801, 1261, 2181, 3041, and 3781) in the AY651061 sequence spanning from the core to the NS3 regions (Fig. 2). Maximum likelihood tree reconstruction based on the nucleotide fragments between the identified breakpoints finally showed that the proposed clustering was confirmed phylogenetically (Fig. 3). AY651061 clusters with HCV subtype 1a in the 5’UTR/core (partial) region (Fig. 3A), the E1 (partial)/E2 (partial) region (Fig. 3C) and the NS2 (partial)/NS3 (partial) region (Fig. 3E). On the other hand, the AY651061 strain is more similar to the HCV 1c subtypes in the core (partial)/E1 (partial) region (Fig. 3B), the E2 (partial)/NS2 (partial) region (Fig. 3D), and the NS3 (partial)/3’UTR region (Fig. 3F). Our findings indicate that AY651061 should be considered as a complex mosaic genome which consists of stretches of nucleotides that belong to both HCV 1a and 1c strains.

## Discussion

To our knowledge, this is the first report on a putative HCV full-length sequence with multiple breakpoints. The interpretation of the findings reported in this communication, like the conclusions drawn in most comparable studies on other members of the family flaviviridae (Holmes et al. 1999; Worobey et al. 1999; Worobey and Holmes, 2001; Twiddy and Holmes, 2003), was evidently based on scrutinising information deposited in the GenBank database by the use of biomathematical tools. The details on the strategy of genome sequencing and cloning of AY651061 available from patent US7348011B2 on the one hand show that the size and location of the individual PCR fragments utilised to generate the full-length sequence do not correspond to the recombination breakpoints identified by our investigation. This observation, in conjunction with the fact that several clones of both the subgenomic fragments and the entire genome were analysed by R.V. Guntaka and co-workers, strongly argue against the consideration that the mosaic structure of AY651061 was simply the result of sequencing errors involving a contaminated or multiple infected sample (Meyerhans et al. 1990; Odelberg et al. 1995). On the other hand, we could not entirely exclude this possibility since we did not have direct access to the original material containing the Khajal HCV isolate, therefore preventing us from further molecular analyses like the use of HCV 1a and 1c subtype-specific oligonucleotide primers spanning the identified recombination breakpoints.

The AY651061 sequence has already been described in a report on HCV recombination published by Cristina and Colina (2006). These authors, however, included AY651061 as a non-recombinant reference sequence in their SimPlot analyses and, therefore, their impression that the query sequence D10749 is a 1a/1c recombinant form with breakpoints in the E1/E2 regions now has to be put under question. A similar classification artefact due to the inclusion of recombinants as reference sequence has been identified for HIV-1 (Abecasis et al. 2007). Interestingly, the putative breakpoints that we identified in AY651061 were almost evenly distributed over the first 4,000 nucleotides of the genome, covering the core to NS3 regions. Thus, these recombination events were not located predominantly in the NS2/NS3 (Kalinina et al. 2002; Kageyama et al. 2006; Noppornpanth et al. 2006; Legrand-Abravanel et al. 2007) or the NS5 regions (Colina et al. 2004; Moreno et al. 2006) that had been described previously as the most likely sites for the occurrence of HCV recombination events.

The molecular structure of AY651061, as revealed by our study, is highly reminiscent of findings in full-length sequences of another member of the genus Flavivirus, i.e. GB virus C (GBV-C), a pathogen closely related to HCV (Simons et al. 1995). In GBV-C, numerous homologous recombinations were detected, leading to the formation of genomes with a rather complex mosaic composition (Twiddy and Holmes, 2003). Worobey and Holmes (2001), for instance, reported on three such GBV-C sequences, one of which showed signs of no less then nine apparent recombination events involving genetic material from at least four different sources and three GBV-C subtypes. Besides the observations in GBV-C sequences, multiple recombinations were also detected in a number of mosquito-borne flaviviruses, among them different serotypes of dengue virus (Holmes et al. 1999; Worobey et al. 1999; Tolou et al. 2001; Twiddy and Holmes, 2003), Japanese encephalitis virus (Twiddy and Holmes, 2003; Gould et al. 2004), and St. Louis encephalitis virus (Twiddy and Holmes, 2003).

Our first observation on a complex mosaic genome pattern in HCV, has to be confirmed and extended by further reports. In the light of the ever increasing amount of HCV sequences available in publically accessible databases, the growing awareness of the possibility of HCV recombination, and the advent of more powerful biomathematical tools that facilitate the detection and characterisation of multiple recombination events (Worobey and Holmes, 1999), we are confident that more sequences will eventually be added to the list of complex mosaic HCV genomes. However, they will probably remain a limited fraction of all available HCV genome sequences. Undoubtedly, the recognition of additional multiple recombinant HCV forms will raise numerous new questions related to HCV research and may also lead to a reconsideration of the current concept of HCV genotyping which is essentially based on the intrinsic assumption that the genotype and subtype assignment inferred from one region also holds true for the genome as a whole (Nolte, 2001; Simmonds et al. 2005; Weck, 2005).
